# Last resort antibiotherapy in sepsis with MDR bacteria, one possible solution in a critical patient (case report)

**DOI:** 10.1186/1471-2334-13-S1-P27

**Published:** 2013-12-16

**Authors:** Doina Iovănescu, Cătălin Apostolescu, Cleo Roşculeț, Monica Ivan, Alina Frangulea, Monica Ungureanu, Ana-Maria Petrescu, Bogdana Manu, Cornel Camburu, Andrei Rogoz, Aida Răşcanu, Marius Radu

**Affiliations:** 1 National Institute for Infectious Diseases “Prof. Dr. Matei Balş”, Bucharest, Romania

## Background

The purpose of this case report was to present the extreme difficulties in finding a solution for the patient with sepsis treatment determined by bacteria associations: MRSA, ESBL *K pneumoniae*, *E coli* with ESBL.

## Case report

We present the case report of a 68-year old male patient, with a history of prostate cancer surgery, relapsed and irradiated. Microbiological and antibiogram data, and the parameters of organ dysfunction represented the basis for an “extreme” decision in the antibiotherapy scheme. We obtained microbiological control and we successfully managed the vital dysfunctions.

## Conclusion

Desperate situations sometimes require last resort decisions, extremely costly which can save the patient for the moment; the question we ask ourselves is whether this control is also supported and for how long.

